# Renal function alters the association of lipoprotein(a) with cardiovascular outcomes in patients undergoing percutaneous coronary intervention: a prospective cohort study

**DOI:** 10.1093/ckj/sfae032

**Published:** 2024-02-09

**Authors:** Guyu Zeng, Pei Zhu, Deshan Yuan, Peizhi Wang, Tianyu Li, Qinxue Li, Jingjing Xu, Xiaofang Tang, Ying Song, Yan Chen, Ce Zhang, Sida Jia, Ru Liu, Lin Jiang, Lei Song, Runlin Gao, Yuejin Yang, Xueyan Zhao, Jinqing Yuan

**Affiliations:** Department of Cardiology, National Clinical Research Center for Cardiovascular Diseases, State Key Laboratory of Cardiovascular Disease, Fuwai Hospital, National Center for Cardiovascular Diseases, Chinese Academy of Medical Sciences and Peking Union Medical College, Beijing, China; Department of Cardiology, National Clinical Research Center for Cardiovascular Diseases, State Key Laboratory of Cardiovascular Disease, Fuwai Hospital, National Center for Cardiovascular Diseases, Chinese Academy of Medical Sciences and Peking Union Medical College, Beijing, China; Department of Cardiology, National Clinical Research Center for Cardiovascular Diseases, State Key Laboratory of Cardiovascular Disease, Fuwai Hospital, National Center for Cardiovascular Diseases, Chinese Academy of Medical Sciences and Peking Union Medical College, Beijing, China; Department of Cardiology, National Clinical Research Center for Cardiovascular Diseases, State Key Laboratory of Cardiovascular Disease, Fuwai Hospital, National Center for Cardiovascular Diseases, Chinese Academy of Medical Sciences and Peking Union Medical College, Beijing, China; Department of Cardiology, National Clinical Research Center for Cardiovascular Diseases, State Key Laboratory of Cardiovascular Disease, Fuwai Hospital, National Center for Cardiovascular Diseases, Chinese Academy of Medical Sciences and Peking Union Medical College, Beijing, China; Department of Cardiology, National Clinical Research Center for Cardiovascular Diseases, State Key Laboratory of Cardiovascular Disease, Fuwai Hospital, National Center for Cardiovascular Diseases, Chinese Academy of Medical Sciences and Peking Union Medical College, Beijing, China; Department of Cardiology, National Clinical Research Center for Cardiovascular Diseases, State Key Laboratory of Cardiovascular Disease, Fuwai Hospital, National Center for Cardiovascular Diseases, Chinese Academy of Medical Sciences and Peking Union Medical College, Beijing, China; Department of Cardiology, National Clinical Research Center for Cardiovascular Diseases, State Key Laboratory of Cardiovascular Disease, Fuwai Hospital, National Center for Cardiovascular Diseases, Chinese Academy of Medical Sciences and Peking Union Medical College, Beijing, China; Department of Cardiology, National Clinical Research Center for Cardiovascular Diseases, State Key Laboratory of Cardiovascular Disease, Fuwai Hospital, National Center for Cardiovascular Diseases, Chinese Academy of Medical Sciences and Peking Union Medical College, Beijing, China; Department of Cardiology, National Clinical Research Center for Cardiovascular Diseases, State Key Laboratory of Cardiovascular Disease, Fuwai Hospital, National Center for Cardiovascular Diseases, Chinese Academy of Medical Sciences and Peking Union Medical College, Beijing, China; Department of Cardiology, National Clinical Research Center for Cardiovascular Diseases, State Key Laboratory of Cardiovascular Disease, Fuwai Hospital, National Center for Cardiovascular Diseases, Chinese Academy of Medical Sciences and Peking Union Medical College, Beijing, China; Department of Cardiology, National Clinical Research Center for Cardiovascular Diseases, State Key Laboratory of Cardiovascular Disease, Fuwai Hospital, National Center for Cardiovascular Diseases, Chinese Academy of Medical Sciences and Peking Union Medical College, Beijing, China; Department of Cardiology, National Clinical Research Center for Cardiovascular Diseases, State Key Laboratory of Cardiovascular Disease, Fuwai Hospital, National Center for Cardiovascular Diseases, Chinese Academy of Medical Sciences and Peking Union Medical College, Beijing, China; Department of Cardiology, National Clinical Research Center for Cardiovascular Diseases, State Key Laboratory of Cardiovascular Disease, Fuwai Hospital, National Center for Cardiovascular Diseases, Chinese Academy of Medical Sciences and Peking Union Medical College, Beijing, China; Department of Cardiology, National Clinical Research Center for Cardiovascular Diseases, State Key Laboratory of Cardiovascular Disease, Fuwai Hospital, National Center for Cardiovascular Diseases, Chinese Academy of Medical Sciences and Peking Union Medical College, Beijing, China; Department of Cardiology, National Clinical Research Center for Cardiovascular Diseases, State Key Laboratory of Cardiovascular Disease, Fuwai Hospital, National Center for Cardiovascular Diseases, Chinese Academy of Medical Sciences and Peking Union Medical College, Beijing, China; Department of Cardiology, National Clinical Research Center for Cardiovascular Diseases, State Key Laboratory of Cardiovascular Disease, Fuwai Hospital, National Center for Cardiovascular Diseases, Chinese Academy of Medical Sciences and Peking Union Medical College, Beijing, China; Department of Cardiology, National Clinical Research Center for Cardiovascular Diseases, State Key Laboratory of Cardiovascular Disease, Fuwai Hospital, National Center for Cardiovascular Diseases, Chinese Academy of Medical Sciences and Peking Union Medical College, Beijing, China; Department of Cardiology, National Clinical Research Center for Cardiovascular Diseases, State Key Laboratory of Cardiovascular Disease, Fuwai Hospital, National Center for Cardiovascular Diseases, Chinese Academy of Medical Sciences and Peking Union Medical College, Beijing, China

**Keywords:** coronary artery disease, estimated glomerular filtration rate, lipoprotein(a), renal function

## Abstract

**Background and hypothesis:**

Lipoprotein(a) [Lp(a)] and renal dysfunction are both independent risk factors for cardiovascular disease. However, it remains unclear whether renal function mediates the association between Lp(a) and cardiovascular outcomes in patients undergoing percutaneous coronary intervention (PCI).

**Methods:**

From a large prospective cohort study, 10 435 eligible patients undergoing PCI from January 2013 to December 2013 were included in our analysis. Patients were stratified into three renal function groups according to their baseline estimated glomerular filtration rate (eGFR) (<60; 60–90; ≥90 ml/min/1.73 m^2^). The primary endpoint was a composite of all-cause death, nonfatal MI, ischemic stroke, and unplanned revascularization [major adverse cardiac and cerebrovascular events (MACCE)].

**Results:**

Over a median follow-up of 5.1 years, a total of 2144 MACCE events occurred. After multivariable adjustment, either eGFR <60 ml/min/1.73 m^2^ or elevated Lp(a) conferred a significantly higher MACCE risk. Higher Lp(a) was significantly associated with an increased risk of MACCE in patients with eGFR <60 ml/min/1.73 m^2^. However, this association was weakened in subjects with only mild renal impairment and diminished in those with normal renal function. A significant interaction for MACCE between renal categories and Lp(a) was observed (*P *= 0.026). Patients with concomitant Lp(a) ≥30 mg/dl and eGFR <60 ml/min/1.73 m^2^ experienced worse cardiovascular outcomes compared with those without.

**Conclusion:**

The significant association between Lp(a) and cardiovascular outcomes was mediated by renal function in patients undergoing PCI. Lp(a)-associated risk was more pronounced in patients with worse renal function, suggesting close monitoring and aggressive management are needed in this population.

KEY LEARNING POINTS **What was known:**

- Patients with coronary artery disease (CAD) remain at a high risk of recurrent ischemic events.
- High lipoprotein(a) [Lp(a)] and renal dysfunction are both contributors to cardiovascular disease.
- Lp(a) levels are inversely correlated with renal function.

**This study adds:**

- Based on a large prospective cohort of CAD patients, we found that the association between Lp(a) and cardiovascular risk diminished in patients with estimated glomerular filtration rate (eGFR)≥90 ml/min/1.73 m², whereas this association became more pronounced in those with worse renal function.
- Patients with both Lp(a)>30 mg/dl and eGFR<60 ml/min/1.73 m² suffered the highest cardiovascular risk among all the participants.

**Potential impact:**

- Concomitant measurement of renal function is warranted for better risk stratification in CAD patients with elevated Lp(a).
- Subjects with worse renal function may derive the greatest benefit from the potential Lp(a) reducing therapy.

## INTRODUCTION

Although evidence-based strategies including revascularization and optimal medical therapy are widely used in clinical practice, patients with coronary artery disease (CAD) remain at a high risk of recurrent ischemic events [1]. Therefore, identification of residual cardiovascular risk is essential for optimizing the outcomes in this population. Lipoprotein(a) [Lp(a)] is a low-density lipoprotein (LDL)-like lipoprotein particle covalently linked to apolipoprotein(a) [apo(a)] and is predominantly genetically determined [2]. Numerous epidemiologic and genetic evidence supports the causal role of Lp(a) in the development of cardiovascular diseases (CVD) [3, 4] Despite the absence of specific Lp(a)-lowering therapies, sub-analyses of randomized trials on the proprotein convertase subtilisin/kexin type 9 (PCSK9) inhibitors suggested the potential benefit of reducing Lp(a) in event reduction secondary to lowering low-density lipoprotein cholesterol (LDL-C) [5, 6]. Hence, Lp(a) might be a potential therapeutic target for further reduction of residual risks.

Chronic kidney disease (CKD) emerges as an important contributor to the global burden of disease. As glomerular filtration rate (GFR) decreases, the prevalence of CVD rises in parallel with the increasing risk of cardiac mortality [7]. Therefore, patients with both CAD and renal dysfunction should be well managed for their high residual cardiovascular risk. Renal function is known to be one of the few influencing factors of Lp(a) concentration irrespective of LPA gene variants [8]. However, whether renal function could influence the association of Lp(a) with cardiovascular risk in CAD patients is not well understood. Our study aimed to investigate the prognostic impact of Lp(a) across varying levels of renal function and the joint effect of Lp(a) and renal dysfunction with cardiovascular outcomes in CAD patients undergoing percutaneous coronary intervention (PCI), based on a large, prospective cohort.

## MATERIALS AND METHODS

### Study population

This is a *post hoc* analysis based on a large prospective observational cohort, which included 10 724 adults undergoing PCI enrolled consecutively in Fuwai Hospital (National Center for Cardiovascular Diseases in China) from January 2013 to December 2013. The indications for PCI were visually estimated diameter stenosis ≥70% (≥50% for left main CAD) and unacceptable angina despite optimal medical therapy [9]. A total of 289 patients missing baseline creatinine and Lp(a) data were excluded, leaving a total of 10 435 eligible patients of East Asian ethnicity in this study.

Our study complied with the Declaration of Helsinki and was approved by the Ethical Review Board of Fuwai Hospital. Written informed consent was signed by all the participants.

### Data collection and definitions

Data on demographic characteristics, laboratory measurements, and medication use were collected from electronic medical records by independent clinical research coordinators. Angiographic data were collected from postoperative reports. Synergy between PCI with TAXUS and Cardiac Surgery (SYNTAX) score was assessed based on coronary lesion details using an online calculator (http://www.syntaxscore.com). Fasting serum samples were gathered routinely in the morning within 24 hours after admission and were tested centrally in the laboratory department of our hospital. Lp(a) was measured by the immunoturbidimetry method [LASAY lipoprotein(a) auto; SHIMA laboratories Co., Ltd] with the recommended threshold of 30 mg/dl [2]. Other lipid parameters such as total cholesterol (TC), triglyceride (TG), LDL-C and high-density lipoprotein cholesterol (HDL-C) were measured by an automated biochemical analyzer (7150; Hitachi, Tokyo, Japan). High-sensitive C-reactive protein (hsCRP) was assessed by immunoturbidimetry (Array 360, Beckman Coulter, Brea, CA, USA). Estimated glomerular filtration rate (eGFR) was calculated based on the Chronic Kidney Disease Epidemiology Collaboration creatinine equation and was regarded as the indicator of renal status [10]. As albumin to creatinine ratio (ACR) was not available in our study, we defined albuminuria as baseline urinary albumin concentration (UAC) ≥20 mg/l according to Kidney Disease: Improving Global Outcomes [11]. Population was divided into three groups by baseline eGFR levels according to the National Kidney Foundation classification: normal renal function (≥90 ml/min/1.73 m^2^), mild renal dysfunction (60–90 ml/min/1.73 m^2^), and moderate to severe renal dysfunction (<60 ml/min/1.73 m^2^) [12]. No patients with eGFR <15 ml/min/1.73 m^2^ were included in this study. Diagnoses of diabetes and hypertension were in accordance with the current guidelines [13, 14]. Diabetes mellitus was diagnosed by self-reported diabetes history, currently receiving antidiabetic medication, HbA1c ≥6.5%, fasting plasma glucose ≥7.0 mmol/L, or two-hour blood glucose of oral glucose tolerance test ≥11.1 mmol/L. Hypertension was defined by hypertension history in medical records with the use of antihypertension drugs, or consecutive systolic blood pressure ≥140 mmHg or/and diastolic blood pressure ≥90 mmHg during hospitalization.

### Follow-up and endpoints

Follow-up data were collected through telephone interviews, correspondence, or clinical visits by well-trained investigators blinded to baseline data at 1 month, 2 months, 1 year, 2 years, and 5 years after discharge. The primary endpoint was major adverse cardiac and cerebrovascular events (MACCE), defined as a composite of all-cause death, nonfatal myocardial infarction (MI), ischemic stroke, and unplanned revascularization. Nonfatal MI was defined by signs or symptoms of newly developed myocardial ischemia with increased cardiac enzyme above the upper reference limit [15]. Ischemic stroke was defined by related symptoms and deficits in neurological imaging. Unplanned revascularization included any repeat PCI or bypass surgery regardless of target segments driven by ischemic symptoms or events. All events were checked and verified centrally by two independent clinicians.

### Statistical analysis

Continuous variables are expressed as mean and standard deviations or median (interquartile range) for non-normally distributed data. Categorical variables are expressed as numbers and percentages. Differences among groups were compared using one-way ANOVA, Kruskal–Wallis H test, or Pearson chi-squared test as appropriate. Cumulative incidences are illustrated by Kaplan–Meier curves and were compared by log-rank tests. Lp(a) was calculated either as categorical variables stratified according to 30 mg/dl as threshold or tertiles or as continuous variables in logarithm form given its skewed distribution. Univariable and multivariable Cox proportional hazard models were applied to assess the hazard ratios for MACCE risk. Most of the variables in Table 1 were included, except angiographic and medication data. The backward stepwise model selection with Akaike information criterion (AIC) was performed to determine the covariates for the multivariable model. After selection, covariates in the multivariable model include age, sex, cardiovascular disease (CVD) history (including prior MI and prior ischemic stroke), hypertension, diabetes, left ventricular ejection fraction <40%, hsCRP, LDL-C, interaction test between Lp(a) and eGFR was used to detect the modification effect. Restricted cubic splines (RCS) analyses with three knots at the fifth, 50th, and 95th centiles were applied to assess the nonlinear relationships between continuous Lp(a) and eGFR and the risk of MACCE. Statistical significance was defined as two-tailed *P *< 0.05. For sensitive analysis, we compared the association between Lp(a) tertiles and MACCE risk between patients with and without albuminuria in 8884 patients with available UAC and 10 318 patients with available eGFR post-procedure. All analyses were performed by R version 4.2.0 (R Foundation for Statistical Computing, Vienna, Austria).

**Table 1:** Baseline characteristics across eGFR groups.

	eGFR ≥ 90 (*n* = 6441)	60 ≤ eGFR < 90 (*n* = 3627)	eGFR < 60 (*n* = 427)	*P* value
Age, yrs	54.5 ± 8.8	64.0 ± 9.3	69.6 ± 9.1	<0.001
Sex, %	1241 (19.3)	964 (27.0)	176 (41.2)	<0.001
BMI, kg/ml	26.0 ± 3.2	25.8 ± 3.2	26.0 ± 3.4	0.001
Prior MI	1214 (18.8)	668 (18.7)	120 (28.1)	<0.001
Prior stroke	527 (8.2)	491 (13.8)	92 (21.5)	<0.001
CAD family history	1744 (27.1)	756 (21.2)	86 (20.1)	<0.001
Diabetes	2670 (41.9)	1584 (45.0)	245 (58.2)	<0.001
Hypertension	3861 (59.9)	2508 (70.3)	352 (82.4)	<0.001
Current smoker	3936 (61.1)	1823 (51.1)	198 (46.4)	<0.001
Clinical presentation				0.002
ACS	3823 (59.4)	2141 (60.0)	291 (68.1)	
CCS	2618 (40.6)	1486 (40.0)	136 (31.9)	
LVEF < 40%	53 (0.8)	46 (1.3)	22 (5.3)	<0.001
LM/three-vessel disease	2660 (41.3)	1761 (49.4)	229 (53.6)	<0.001
SYNTAX score*	10 (6–16)	11 (6–17)	12 (6–21)	<0.001
Calcification	94 (2.5)	88 (4.1)	12 (4.1)	0.001
Number of stents	1.8 ± 1.1	1.8 ± 1.1	1.8 ± 1.2	0.536
TG, mmol/l	1.55 (1.16–2.14)	1.49 (1.11–2.03)	1.59 (1.23–2.16)	<0.001
LDL-C, mmol/l	2.35 (1.86–3.00)	2.34 (1.86–3.01)	2.37 (1.84–2.98)	0.848
HDL-C, mmol/l	0.98 (0.83–1.16)	1.02 (0.86–1.20)	0.98 (0.81–1.15)	<0.001
TC, mmol/l	4.04 (3.44–4.81)	4.07 (3.45–4.80)	4.03 (3.42–4.82)	0.921
eGFR, ml/min/1.73 m^2^	99.7 (95.2–105.3)	80.9 (73.7–86.3)	52.9 (46.8–56.6)	<0.001
Lipoprotein (a), mg/dl	178.2 (74.4–406.2)	191.5 (83.6–419-3)	217.8 (96.0–485.9)	0.001
hsCRP, mg/l	1.52 (0.76–3.41)	1.71 (0.86–3.92)	3.14 (1.30–9.00)	<0.001
Medication at discharge				
Statin	6184 (96.0)	3421 (95.9)	405 (94.8)	0.499
Aspirin	6367 (98.9)	3523 (98.8)	414 (97.0)	0.003
Clopidogrel	6338 (98.4)	3522 (98.7)	420 (98.4)	0.395
CCB	3061 (47.5)	1813 (50.8)	206 (48.2)	0.007
β-blocker	5827 (90.5)	3204 (89.8)	383 (89.7)	0.545

ACS, acute coronary syndrome; BMI, body mass index; CAD, coronary artery disease; CCB, calcium channel blocker; CCS, chronic coronary syndrome; DES, drug-eluting stent; eGFR, estimated glomerular filtration rate; HDL-C, high-density lipoprotein cholesterol; hsCRP, high-sensitive C-reactive protein; LDL-C, low-density lipoprotein cholesterol; LM, left main artery; LVEF, left ventricular ejection fraction; MI, myocardial infarction; SYNTAX, SYNergy between PCI with TAXUS and Cardiac Surgery; TC, total cholesterol; TG, triglyceride.

* SYNTAX score was calculated using an online calculator (http://www.syntaxscore.com) by a research group blinded to the clinical data.

## RESULTS

### Baseline characteristics of study population

A total of 10 435 patients meeting inclusion and exclusion criteria were enrolled, of which 6441 (61.7%) had normal renal function (eGFR ≥90 ml/min/1.73 m^2^), 3567 (34.2%) had mildly impaired renal function (60 ≤ eGFR<90 ml/min/1.73 m^2^), and 427 (4.1%) had moderate to severe renal dysfunction (eGFR <60 ml/min/1.73 m^2^). The mean age of the included patients was 58.4 ± 10.3 years (22.8% were women). Median eGFR and Lp(a) were 94.1 ml/min/1.73 m^2^ and 185.4 mg/dl, respectively. Baseline characteristics across renal function groups are presented in Table 1. Patients with impaired renal function were older and more likely to be female. They also had higher prevalence of prior MI, smoking, ACS, LVEF <40%, complex CAD, and comorbidities including diabetes and hypertension, with higher levels of Lp(a) and hsCRP.

### Renal function, lipoprotein(a), and cardiovascular outcomes

**Figure 1:**
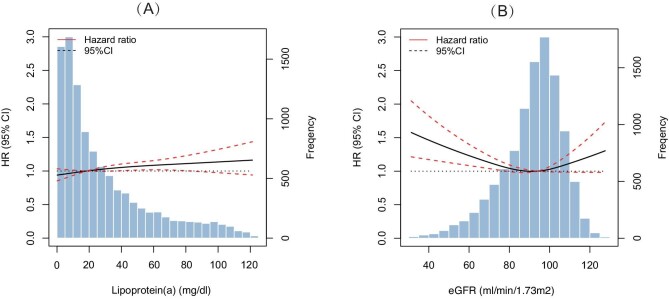
Association between estimated glomerular filtration rate (eGFR) and lipoprotein(a) [Lp(a)] and MACCE risk on continuous scale. MACCE, major adverse cardiac and cerebrovascular events.

During a median 5.1-year follow-up period, 2144 (20.5%) MACCE events (including 375 all-cause deaths, 225 cardiac deaths, 560 nonfatal MIs, 311 strokes, and 1390 unplanned revascularizations) occurred. Patients with high Lp(a) or low eGFR levels experienced a higher prevalence of MACCE (Table 2). After multivariable adjustment, higher Lp(a) on both continuous and categorical scales were significantly associated with higher MACCE risks (≥30 vs. <30 mg/dl: HR 1.12, 95%CI 1.03–1.23; logarithm form per 1 unit: HR 1.11, 95%CI 1.01–1.21), whereas the significant associations with higher risks of MACCE were only observed in patients with eGFR<60 ml/min/1.73 m² (<60 vs. ≥90 ml/min/1.73 m^2^: HR 1.31, 95%CI 1.08–1.60). According to Fig. 1, the association between Lp(a) and MACCE was linear, but the risk pattern between eGFR and MACCE was U-shaped.

**Table 2:** Lipoprotein(a), renal function and MACCE events.

	Event/total (%)	Crude HR (95%CI)	*P* value	Adjusted HR (95%CI)	*P* value
Lp(a) (mg/dl)					
Lg(Lp(a)) per 1 unit	2144/10 435 (20.5)	1.14 (1.04–1.24)	0.004	1.11 (1.01–1.21)	0.022
Lp(a) <30	1336/6799 (19.6)	Reference		Reference	
Lp(a) ≥30	808/3636 (22.2)	1.15 (1.06–1.26)	0.001	1.12 (1.03–1.23)	0.011
eGFR (ml/min/1.73 m²)					
eGFR per 10 unit	2144/10 435 (20.5)	0.92 (0.90–0.95)	<0.001	0.97 (0.94–1.00)	0.076
eGFR ≥90	1246/6441 (19.3)	Reference		Reference	
60 ≤ eGFR <90	764/3627 (21.4)	1.12 (1.02–1.22)	0.017	1.00 (0.90–1.11)	0.991
eGFR <60	134/427 (31.4)	1.74 (1.45–2.08)	<0.001	1.31 (1.08–1.60)	0.008

Multivariable models were adjusted for age, sex, cardiovascular disease history, hypertension, diabetes, left ventricular ejection fraction <40%, low-density lipoprotein cholesterol, high-sensitive C-reactive protein.

CI, confidence interval; eGFR, estimated glomerular filtration rate; HR, hazard ratio; Lp(a), lipoprotein(a), MACCE, major adverse cardiac and cerebrovascular events.

### Prognostic impact of lipoprotein(a) across different renal function groups

**Figure 2:**
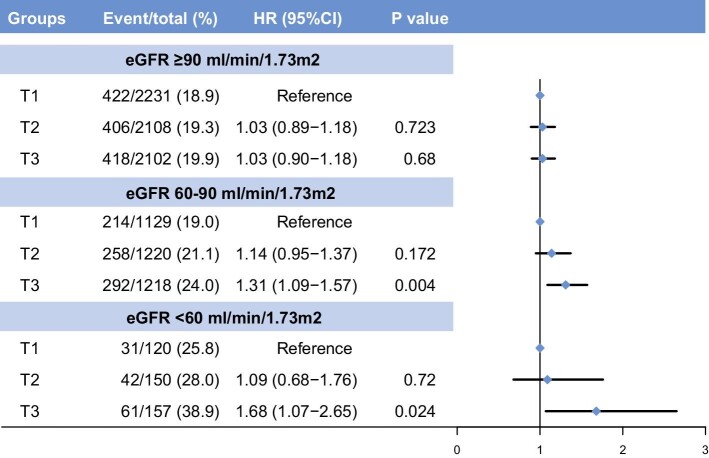
MACCE risk by lipoprotein(a) [Lp(a)] tertiles (<10.7; 10.7–31.4; ≥31.4 mg/dl) and renal function categories. CI, confidence interval; eGFR, estimated glomerular filtration rate; HR, hazard ratio; MACCE, major adverse cardiac and cerebrovascular events.

To further explore the Lp(a)-associated cardiovascular risks across renal function status, patients were stratified into three groups according to their eGFR levels. Patients with increasing Lp(a) (Lp(a) ≥30 mg/dl) had significantly higher MACCE rates in impaired renal function groups (eGFR 60–90 ml/min/1.73 m²: 24.1% vs. 19.9%, *P *= 0.004; eGFR <60 ml/min/1.73 m²: 39.3% vs. 26.5%, *P *= 0.008), but not in those with normal renal function (19.9% vs. 19.1%, *P *= 0.470). After adjusting for covariates, each unit increase of Lp(a) logarithm was significantly associated with higher MACCE risks only in the subjects with eGFR in the range of 60 to 90 ml/min/1.73 m² (HR 1.25, 95%CI 1.07–1.46) (Table 3). In the setting of categorical Lp(a) (≥30 vs. <30 mg/dl), elevated Lp(a) was significantly associated with 23% and 68% increasing risks of MACCE in patients with eGFR 60-90 ml/min/1.73 m² (HR 1.23, 95%CI 1.06–1.43) and <60 ml/min/1.73 m² (HR 1.68, 95%CI 1.18–2.40), respectively. There was a significant interaction for MACCE between dichotomous Lp(a) and eGFR categories (*P *= 0.026). When stratifying Lp(a) levels into tertiles (<10.7; 10.7–31.4; ≥31.4 mg/dl), the risks of MACCE increased in a stepwise manner following increasing Lp(a) tertiles in patients with renal dysfunction and were more pronounced in those with eGFR <60 ml/min/1.73 m² (Fig. 2).

**Table 3:** Lipoprotein(a)-associated cardiovascular risk stratified by renal function.

	Event/total (%)	Crude HR (95%CI)	*P* value	Adjusted HR (95%CI)	*P* value
eGFR≥90 ml/min/1.73 m²					
Lg(Lp(a)) per unit	1246/6441 (19.3)	1.05 (0.94–1.17)	0.394	1.03 (0.92–1.15)	0.670
Lp(a)<30 mg/dl	811/4251 (19.1)	Reference		Reference	
Lp(a)≥30 mg/dl	435/2190 (19.9)	1.05 (0.93–1.18)	0.449	1.02 (0.90–1.15)	0.748
60 ≤ eGFR<90 ml/min/1.73 m²					
Lg(Lp(a)) per unit	764/3567 (21.4)	1.26 (1.09–1.46)	0.002	1.25 (1.07–1.46)	0.005
Lp(a)<30 mg/dl	455/2284 (19.9)	Reference		Reference	
Lp(a)≥30 mg/dl	309/1283 (24.1)	1.25 (1.08–1.45)	0.002	1.23 (1.06–1.43)	0.007
eGFR<60 ml/min/1.73 m²					
Lg(Lp(a)) per unit	134/427 (31.4)	1.27 (0.89–1.82)	0.184	1.26 (0.86–1.84)	0.231
Lp(a)<30 mg/dl	70/264 (26.5)	Reference		Reference	
Lp(a)≥30 mg/dl	64/163 (39.3)	1.62 (1.15–2.27)	0.005	1.68 (1.18–2.40)	0.004

Multivariable models were adjusted for age, sex, cardiovascular disease history, hypertension, diabetes, left ventricular ejection fraction <40%, low-density lipoprotein cholesterol, high-sensitive C-reactive protein.

CI, confidence interval; eGFR, estimated glomerular filtration rate; HR, hazard ratio; Lp(a), lipoprotein(a).

In sensitive analysis, the significant association between Lp(a) tertiles and MACCE risk was more prominent in patients with albuminuria (UAC ≥20 mg/l) (Table S1, see online supplementary material). However, this effect is not shown in the normal renal function group (Table S2, see online supplementary material). When stratified by eGFR categories after the procedure, higher Lp(a) were significantly associated with elevated MACCE risk only in patients with eGFR <60 ml/min/1.73 m² (Table S3, see online supplementary material).

### Combination of lipoprotein(a) and renal function

**Figure 3:**
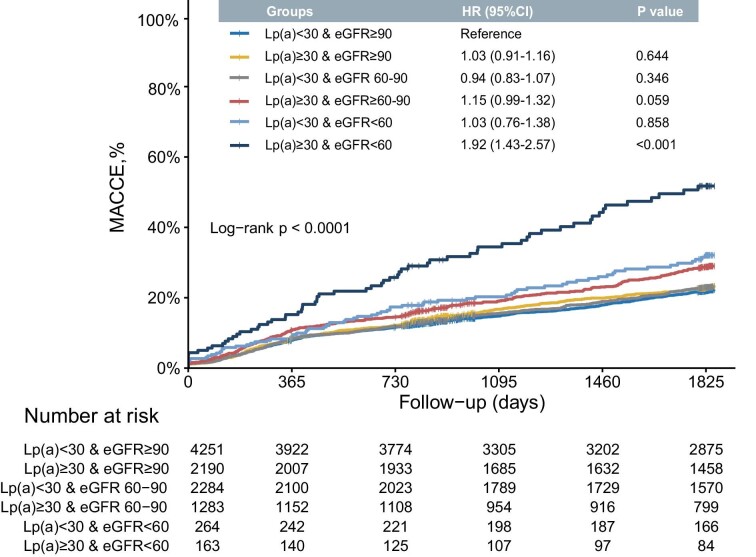
Joint effect of renal function and lipoprotein(a) [Lp(a)] on predicting MACCE risk. CI, confidence interval; eGFR, estimated glomerular filtration rate; HR, hazard ratio; MACCE, major adverse cardiac and cerebrovascular events.

Fig. 3 depicts the results of multivariable Cox regression analyses and cumulative incidences of MACCE in six groups categorized by eGFR and Lp(a) thresholds. Compared to subjects with eGFR ≥90 ml/min/1.73 m^2^ and Lp(a) <30 mg/dl, only those with both eGFR <60 ml/min/1.73 m^2^ and elevated Lp(a) were associated with a significantly higher risk of MACCE (adjusted HR 1.92, 95%CI 1.43–2.57) and higher cumulative incidence.

### Additional prognostic performance of lipoprotein(a)

Table 4 describes the incremental prognostic performance by adding Lp(a) into the original Cox regression model. Lp(a) (both on continuous and categorical scales) could significantly improve the reclassification of the original model in the entire cohort and both impaired renal function groups. No improvement was seen in individuals with normal renal function.

**Table 4:** Discrimination and reclassification performance of lipoprotein(a).

	C-statistic	*P* value	IDI	*P* value	NRI	*P* value	AIC
Entire cohort							
Original model	0.570		Reference		Reference		37 622.8
Original model + continuous lp(a)	0.570	0.726	<0.001 (0–0.001)	0.029	0.061 (0.015–0.108)	0.010	37 619.9
Original model + categorical lp(a)	0.570	0.628	<0.001 (0–0.001)	0.013	0.071 (0.025–0.118)	0.003	37 618.4
eGFR ≥90							
Original model	0.564		Reference		Reference		20 758.0
Original model + continuous lp(a)	0.564	0.965	0	0.719	−0.019 (−0.079–0.042)	0.540	20 759.9
Original model + categorical lp(a)	0.564	0.988	0	0.859	0.023 (−0.037–0.083)	0.455	20 761.8
eGFR 60–90							
Original model	0.556		Reference		Reference		11 811.8
Original model + continuous lp(a)	0.563	0.206	0.002 (0–0.004)	0.007	0.108 (0.029–0.187)	0.008	11 805.4
Original model + categorical lp(a)	0.563	0.201	0.002 (0–0.004)	0.011	0.113 (0.033–0.192)	0.005	11 807.4
eGFR <60							
Original model	0.577		Reference		Reference		1507.0
Original model + continuous lp(a)	0.605	0.218	0.016 (0.003–0.029)	0.014	0.218 (0.016–0.419)	0.034	1501.8
Original model + categorical lp(a)	0.614	0.143	0.018 (0.005–0.032)	0.007	0.284 (0.080–0.488)	0.006	1504.3

AIC, Akaike's information Criterion; eGFR, estimated glomerular filtration rate; IDI, integrated discrimination improvement; Lp(a), lipoprotein(a); NRI, net reclassification improvement.

## DISCUSSION

In this large prospective cohort study, we demonstrated a significant interaction between Lp(a) and renal function in patients undergoing PCI. Elevated Lp(a) and renal dysfunction with eGFR <60 ml/min/1.73 m² were both significantly associated with MACCE risk in the total population. However, the association between Lp(a) and MACCE risk diminished in patients with eGFR ≥90 ml/min/1.73 m² whereas this association became more pronounced in those with worse renal function. Similarly, Lp(a) improved the reclassification of the original model only in patients with renal impairment (eGFR <90 ml/min/1.73 m²), but not in those with normal renal function. Patients with both Lp(a) >30 mg/dl and eGFR <60 ml/min/1.73 m² suffered the highest cardiovascular risk among all the participants.

Lp(a) is well recognized as a substantial causal risk factor for CVD. Lp(a) composition contributes to atherosclerosis, calcification, thrombosis, and vascular inflammation, which further promotes disease development and progression [16]. Hence, Lp(a) measurement was recommended at least once in one's lifetime to identify cardiovascular risk by the recent European Society of Cardiology guideline [17]. Concerning secondary prevention, elevated Lp(a) was also found to be associated with a higher risk of MACCE. In a large meta-analysis, CAD patients with the highest Lp(a) quantile conferred a 40% increased risk of cardiac event [18]. Similarly, several cohort studies and secondary analyses of clinical trials reported a positive association between Lp(a) and adverse outcomes in patients with established CAD or receiving PCI [19–22]. In our study, the adjusted risk of MACCE was significantly higher in patients with elevated Lp(a) levels, which was in accordance with previous studies.

The prevalence of CKD increased continuously in the past few decades, with around 10% of the worldwide population being affected [23]. CKD is an independent risk factor for CVD. Furthermore, even mild renal dysfunction (GFR 60–90 ml/min/1.73 m²) is associated with an increased risk of developing CVD [24]. In addition to the traditional cardiovascular risk factors such as hypertension and diabetes mellitus, dyslipidemia related to renal dysfunction also contributes significantly to excess comorbidity of CVD and even cardiac mortality in these patients [25]. Impaired renal function alters lipid metabolism and changes lipid level and structure, inducing a more atherogenic profile [26]. Elevated Lp(a) levels are a notable manifestation of dyslipidemia specific to renal dysfunction, which was also shown in our study [8]. Previous studies reported that the inverse correlation between renal function and Lp(a) concentration is influenced by factors such as ethnicity, CKD severity, and apo(a) phenotype [27, 28]. Based on the measurement of Lp(a) plasma levels in arteries and in renal veins, Kronenberg *et al*. suggested that Lp(a) elevation could be a result of the impaired Lp(a) clearance by the kidneys [29]. Further studies are warranted to better investigate the relationship between Lp(a) and renal impairment.

Although the causal association of Lp(a) with cardiovascular risk is fully understood, the interrelationship between Lp(a), cardiovascular outcomes, and renal dysfunction, particularly in patients with mild renal impairment, is not determined. Previous studies on this issue produced inconsistent results, potentially due to the heterogeneity across studies such as differences in ethnicity, apo(a) isoform sizes, sample sizes, and endpoints. Analysis of 3939 CKD patients from Chronic Renal Insufficiency Cohort (CRIC) study found that increasing Lp(a) levels were independently associated with the risk of MI and death [30]. In the 4D study, a significant association between Lp(a) and death risk was observed in 1225 hemodialysis patients with diabetes [31]. Notably, this association was largely driven by infection-related death, not cardiac death. According to the Cardiovascular Health Study based on 5808 dwellers from four American communities, the association between Lp(a) and cardiac death was significant in individuals without CKD and close to significance in those with CKD [32]. Furthermore, high-quality evidence is lacking in the setting of secondary cardiovascular prevention. Two studies with small sample sizes reported that elevated Lp(a) was significantly associated with a higher risk of adverse outcomes in CKD patients undergoing PCI [33, 34]. In a recent study of 1306 ACS patients conducted by Li *et al*., the association between Lp(a) and risk of major adverse clinical events was significant and consistent in both CKD and non-CKD groups [35]. Conversely, another cohort study including 51 500 CAD patients found that only patients with eGFR<60 ml/min/1.73 m² suffered from increased adjusted risk for all-cause death associated with elevated Lp(a), with a significant interaction between eGFR groups and Lp(a) levels [36]. Similarly, Yoon *et al*. also demonstrated that the association between Lp(a) and primary outcomes was more pronounced in patients with CKD among 12 064 patients undergoing PCI [21]. However, none of these studies verified the prognostic impact of Lp(a) in patients in the range of 60 to 90 ml/min/1.73 m². Based on a large prospective cohort, we found that the association between Lp(a) and MACCE risk was significant in PCI patients with renal dysfunction, both with eGFR<60 ml/min/1.73 m² and eGFR 60–90 ml/min/1.73 m². The possible underlying mechanisms of our findings could be explained by the amplified pro-inflammatory and pro-calcifying effects of Lp(a) (mainly by oxidized phospholipids) in CKD, as CKD also induces a pro-inflammatory state and vascular calcification, which are vital risk factors for cardiovascular disease [8, 37]. Additionally, since Lp(a) is considered to accelerate renal injury and CKD could lead to increased Lp(a) levels, this interaction may worsen renal function and further increase the risk of recurrent cardiovascular events [38, 39]. Further studies are needed to verify the mechanistic basis of our observations.

Despite observational studies indicating a potential benefit of Lp(a)-lowering therapy, there is no direct proof for pharmacological treatment currently. PCSK9 inhibitors have been found to modestly reduce Lp(a) levels by 25% to 30% in addition to their LDL-C lowering effect. Secondary analyses from the FOURIER trial and ODYSSEY Outcomes trial revealed that patients with higher baseline Lp(a) levels experienced a greater treatment effect of PCSK9 inhibitors on major adverse outcomes. Moreover, reducing Lp(a) levels was found to be associated with a reduction in major adverse cardiovascular events, although the main beneficial effect of LDL-C reduction was not eliminated. Recently, the use of antisense RNA targeting apo(a) was regarded as a promising approach for controlling Lp(a)-associated cardiovascular risk, as it was shown to achieve a dramatic reduction in Lp(a) [40]. The clinical effectiveness of pelacarsen (an antisense oligonucleotide agent) in reducing major cardiovascular events is being evaluated among 8323 CVD patients in the ongoing Lp(a) HORIZON trial (NCT04023552). In our study, we found that CAD patients with both renal dysfunction and elevated Lp(a) had the highest risk of MACCE. Therefore, it is estimated that these high-risk subjects might derive the greatest benefit from Lp(a) reducing therapy.

Several limitations in our study should be noted. First, considering its single-center prospective cohort design, inherent limitations, and bias were inevitable. Moreover, as Lp(a) level differs across different ethnicities, our results may not extrapolate to other ethnic groups. Second, Lp(a) and creatinine were measured only at a single time point, and thus the potential impact of fluctuations in Lp(a) and creatinine levels over time is unknown. Third, the low proportion of subjects with CKD in stages 3b, 4, and 5 raises concerns about the generalizability of our results to this population. Therefore, our findings should be validated in further studies involving more CKD and dialysis patients. Fourth, data on other indicators such as albumin/creatinine ratio and cystatin C were not routinely collected, which may lead to inaccuracy in determining renal dysfunction. Fifth, although the immune turbidimetry assay is widely used for Lp(a) measurement in clinical practice, Lp(a) levels could potentially be underestimated or overestimated due to the apo(a) isoform-insensitive nature of this method. Sixth, the generalizability of our results to the current clinical landscape is limited, as new drugs demonstrating efficacy in reducing mortality risk among high-risk patients (such as SGLT2 inhibitors) have emerged after enrollment.

## CONCLUSION

In summary, our prospective cohort study demonstrated that the association between Lp(a) and major adverse outcomes was only observed in patients with renal impairment in the setting of secondary prevention. Concomitant measurement of renal function is warranted for better risk stratification in CAD patients with elevated Lp(a).

## Supplementary Material

sfae032_Supplemental_File

## Data Availability

Data in this study are available after reasonable request to the corresponding author.
