# Supplementary material for: Renal function alters the association of lipoprotein(a) with cardiovascular outcomes in patients undergoing percutaneous coronary intervention: a prospective cohort study
Source: Clin Kidney J. 2024 Feb 9;17(3):sfae032. doi: 10.1093/ckj/sfae032 (PMC10906361; doi:10.1093/ckj/sfae032)
Supplement: sfae032_Supplemental_File [file sfae032_supplemental_file.docx]

**Supplementary Materials**

Supplementary Table 1. MACCE risk by lipoprotein(a) tertiles (<10.7; 10.7-31.4; ≥31.4 mg/dl) and urinary albumin concentration.

|  | Event/total (%) | Crude HR (95%CI) | P value | Adjusted HR (95%CI) | P value |
| --- | --- | --- | --- | --- | --- |
| Without albuminuria (n=6,633) |  |  |  |  |  |
| Lp(a) tertile 1 | 418/2193 (19.1) | Reference |  | Reference |  |
| Lp(a) tertile 2 | 433/2224 (19.5) | 1.02 (0.89-1.17) | 0.740 | 1.03 (0.90-1.18) | 0.677 |
| Lp(a) tertile 3 | 483/2216 (21.8) | 1.17 (1.03-1.33) | 0.019 | 1.17 (1.02-1.34) | 0.025 |
| With albuminuria (n=2,251) |  |  |  |  |  |
| Lp(a) tertile 1 | 146/764 (19.1) | Reference |  | Reference |  |
| Lp(a) tertile 2 | 177/746 (23.7) | 1.28 (1.03-1.59) | 0.029 | 1.29 (1.03-1.62) | 0.026 |
| Lp(a) tertile 3 | 189/741 (25.5) | 1.38 (1.12-1.72) | 0.003 | 1.32 (1.06-1.66) | 0.015 |

Multivariable models were adjusted for age, sex, cardiovascular disease history, hypertension, diabetes, left ventricular ejection fraction<40%, low-density lipoprotein cholesterol, high-sensitive C-reactive protein.

Abbreviations: CI, confidence interval; HR, hazard ratio; Lp(a), lipoprotein(a), MACCE, major adverse cardiac and cerebrovascular events, UAC, urinary albumin concentration.

Supplementary Table 2. The association between lipoprotein(a) tertiles (<10.7; 10.7-31.4; ≥31.4 mg/dl) and MACCE risk stratified by albuminuria status and baseline eGFR categories.

| eGFR categories | Albuminuria status | Lp(a) categories | Event/total (%) | Adjusted HR (95%CI) | P value |
| --- | --- | --- | --- | --- | --- |
| eGFR≥90 ml/min/1.73m² | Without albuminuria | Lp(a) tertile 1 | 271/1435 (18.9) | Reference |  |
|  |  | Lp(a) tertile 2 | 269/1387 (19.4) | 1.03 (0.87-1.23) | 0.704 |
|  |  | Lp(a) tertile 3 | 273/1373 (19.9) | 1.05 (0.88-1.25) | 0.564 |
|  | With albuminuria | Lp(a) tertile 1 | 89/453 (19.6) | Reference |  |
|  |  | Lp(a) tertile 2 | 81/402 (20.1) | 1.05 (0.77-1.43) | 0.754 |
|  |  | Lp(a) tertile 3 | 80/386 (20.7) | 0.99 (0.72-1.36) | 0.944 |
| eGFR 60-90 ml/min/1.73m² | Without albuminuria | Lp(a) tertile 1 | 127/702 (18.1) | Reference |  |
|  |  | Lp(a) tertile 2 | 147/763 (19.3) | 1.10 (0.86-1.40) | 0.458 |
|  |  | Lp(a) tertile 3 | 182/776 (23.5) | 1.36 (1.07-1.72) | 0.011 |
|  | With albuminuria | Lp(a) tertile 1 | 48/259 (18.5) | Reference |  |
|  |  | Lp(a) tertile 2 | 74/279 (26.5) | 1.58 (1.09-2.30) | 0.016 |
|  |  | Lp(a) tertile 3 | 83/282 (29.4) | 1.75 (1.21-2.53) | 0.003 |

Multivariable models were adjusted for age, sex, cardiovascular disease history, hypertension, diabetes, left ventricular ejection fraction<40%, low-density lipoprotein cholesterol, high-sensitive C-reactive protein.

Abbreviations: CI, confidence interval; eGFR, estimated glomerular filtration rate, HR, hazard ratio; Lp(a), lipoprotein(a), MACCE, major adverse cardiac and cerebrovascular events.

Supplementary Table 2. MACCE risk by Lp(a) tertiles (<10.7; 10.7-31.4; ≥31.4 mg/dl) and eGFR categories after procedure.

|  | Event/total (%) | Crude HR (95%CI) | P value | Adjusted HR (95%CI) | P value |
| --- | --- | --- | --- | --- | --- |
| eGFR≥90 ml/min/1.73m² (n=4,856) |  |  |  |  |  |
| Lp(a) tertile 1 | 312/1679 (18.6) | Reference |  | Reference |  |
| Lp(a) tertile 2 | 317/1597(19.8) | 1.08 (0.92-1.26) | 0.362 | 1.10 (0.93-1.28) | 0.264 |
| Lp(a) tertile 3 | 311/1580 (19.7) | 1.07 (0.92-1.26) | 0.384 | 1.08 (0.92-1.27) | 0.368 |
| eGFR 60-90 ml/min/1.73m² (n=4,821) |  |  |  |  |  |
| Lp(a) tertile 1 | 315/1588 (19.8) | Reference |  | Reference |  |
| Lp(a) tertile 2 | 321/1617 (19.9) | 1.00 (0.85-1.17) | 0.983 | 0.99 (0.85-1.17) | 0.943 |
| Lp(a) tertile 3 | 360/1616 (22.3) | 1.15 (0.98-1.33) | 0.078 | 1.12 (0.95-1.31) | 0.171 |
| eGFR<60 ml/min/1.73m² (n=641) |  |  |  |  |  |
| Lp(a) tertile 1 | 34/178 (19.1) | Reference |  | Reference |  |
| Lp(a) tertile 2 | 60/224 (26.8) | 1.49 (0.98-2.27) | 0.063 | 1.52 (0.99-2.33) | 0.058 |
| Lp(a) tertile 3 | 85/239 (35.6) | 2.05 (1.38-3.05) | <0.001 | 2.06 (1.36-3.11) | 0.001 |

Multivariable models were adjusted for age, sex, cardiovascular disease history, hypertension, diabetes, left ventricular ejection fraction<40%, low-density lipoprotein cholesterol, high-sensitive C-reactive protein.

Abbreviations: CI, confidence interval; eGFR, estimated glomerular filtration rate, HR, hazard ratio; Lp(a), lipoprotein(a), MACCE, major adverse cardiac and cerebrovascular events.
